# AST·MLR index and operation injury condition are novel prognostic predictor for the prediction of survival in patients with colorectal cancer liver metastases undergoing surgical resection

**DOI:** 10.1186/s12885-022-10009-4

**Published:** 2022-08-26

**Authors:** Qichen Chen, Mingxia Li, Jinghua Chen, Zhen Huang, Xiao Chen, Hong Zhao, Jianqiang Cai

**Affiliations:** 1 Department of Hepatobiliary Surgery, National Cancer Center/National Clinical Research Center for Cancer/Cancer Hospital, Chinese Academy of Medical Sciences and Peking Union Medical College, Beijing, China; 2 Department of Thoracic Surgery, National Cancer Center/National Clinical Research Center for Cancer/Cancer Hospital, Chinese Academy of Medical Sciences and Peking Union Medical College, Beijing, China; 3 Peking Union Medical College, Chinese Academy of Medical Sciences and Peking Union Medical College, Beijing, China

**Keywords:** Colorectal neoplasms, Neoplasm metastasis, Nomograms, Prognosis, Aspartate aminotransferases, Monocytes, Lymphocytes, Operation injury condition

## Abstract

**Background:**

The prognostic values of preoperative aspartate aminotransferase (AST), monocyte-to-lymphocyte ratio (MLR), AST·MLR index (AMLRI) and operation injury condition in patients with colorectal cancer liver metastases (CRLM) remains unclear. This retrospective study assessed the relationship between these markers, progression-free survival (PFS), and overall survival (OS) in CRLM patients undergoing resection.

**Methods:**

AMLRI was defined as AST × MLR. Operation injury condition was defined according to operation time and blood loss. Cox regression analyses were used to identify risk factors and to develop nomograms. C-indexes, time-dependent receiver operating characteristic (time-ROC) curves and calibration curves were used to assess the models.

**Results:**

A total of 379 patients were enrolled. The optimal cut-off value of the AMLRI was 3.33. In the multivariable analysis, AMLRI > 3.33 (hazard ratio [HR] = 2.162, *p* = 0.002) and serious operation injury condition (HR = 1.539, *p* = 0.012) were predictive for unfavourable OS, and AMLRI > 3.33 (HR = 1.462, *p* = 0.021) was predictive for unfavourable PFS. The nomograms were superior to Fong’s Clinical Risk Score (CRS) according to the C-indexes (PFS: 0.682 vs. 0.600; OS: 0.730 vs. 0.586) and time-ROCs.

**Conclusions:**

Preoperative AMLRI and operation injury condition are easily accessible predictors for prognosis. The nomograms performed better than CRS for the prediction of recurrence and survival.

**Supplementary Information:**

The online version contains supplementary material available at 10.1186/s12885-022-10009-4.

## Background

Colorectal cancer (CRC) has become the third most common cancer and the second reason for cancer-related deaths, which metastasizes to liver in more than 50% patients [1]. Hepatectomy is considered the optimal choice of treatment for CRLM. However, the postoperative recurrence rate remains high, ranging from 60 to 80% [2]. Therefore, reliable prognostic markers should be identified to recognize patients at high risk of recurrence early and predict their survival, in order to apply proper adjuvant therapies.

The serum level of aspartate aminotransferase (AST) is routinely used in the test of liver function to assess liver damage. Some studies have indicated that AST may be significantly associated with survival in some kinds of cancers, but not in CRLM [3, 4]. Inflammation is regarded as another key factor in the development of tumours [5]. The monocyte-to-lymphocyte ratio (MLR) is an inflammatory index. However, its prognostic values for CRLM have not been thoroughly investigated, limited in patients with unresectable tumours receiving radiofrequency ablation or patients receiving neoadjuvant therapy [6, 7]. Additionally, several studies have indicated that more blood loss in surgery represents poorer OS after curative surgery [8, 9]. It would be meaningful to evaluate the influence of operation injury condition on the prognosis of CRLM.

In this study, we accessed and compared the prognostic values of AST and MLR, and established the AST·MLR index (AMLRI). In addition, the operation injury condition was assessed based on the operation time and blood loss. Nomograms based on these markers and other risk factors were furtherly established for the prediction of prognosis in patients with CRLM undergoing resection to compared with Fong’s clinical risk score, which is widely used to predict recurrence and survival of CRLM [10].

## Methods

### Patients

Data were retrospectively collected from 389 patients admitted to Cancer Hospital, Chinese Academy of Medical Sciences from April 2012 to December 2018. The inclusion criteria were as follows: 1, the primary tumour was resected and diagnosed as CRC pathologically. 2, the liver site was diagnosed pathologically as CRLM. 3, patients were treated surgically according to the guidelines for the treatment of CRLM [1]. The exclusion criteria were as follows: 1, patients with severe diseases which might affect the AST and MLR, such as severe infections, cardiovascular diseases, hepatitis, etc. 2, patients with multiple primary malignant neoplasms. 3, patients with incomplete data. Existence of extrahepatic metastases of CRC was not a contraindication for surgery and these patients were included, as these sites could be radically treated (surgery, radiotherapy, ablation, etc.). Data, including patient demographics, preoperative haematologic markers and therapeutic strategy and outcomes were collected, which will be described in detail below.

### Specimens

Haematologic examinations, including routine blood tests, liver function tests which include serum AST and serum carcinoembryonic antigen (CEA) tests, were performed within 1 week before surgery. None of the patients presented signs of diseases which might influence AST and MLR at the time of blood collection. Serum AST concentrations were determined by enzyme kinetic assay (Roche Diagnostics GmbH, Mannheim, Germany). The normal AST levels ranging from 15 to 40 U/L at our hospital. Serum CEA concentrations were determined by electrochemiluminescence immunoassay method.

### Markers and definitions

The indexes were calculated according to the following formulas: MLR = (monocyte count/lymphocyte count); AMLRI = AST × MLR. The operation injury condition was defined according to operation time and blood loss as follows: X-tile software (version 3.6.1, Yale University) was used to determine the optimal cut-off value of operation time and blood loss according to survival. According to the results, if operation time > 340 min or blood loss > 400 mL, the patient was defined as having a serious operation injury condition. The cut-off value of preoperative CEA was set at 50 ng/mL which was widely used in previous study [11]. The resection margin status was defined according to the International Union Against Cancer criteria. Major resections were defined as resections of more than two segments of liver. The CRS developed by Fong et al. was calculated and divided into 2 groups: 0–2 and 3–5 [10, 12].

### Treatment

The therapeutic strategy for patients was designed individually after discussion by a multidisciplinary team including surgeons, oncologists (medical and radiation), radiologists and pathologists. Patients with initially unresectable liver metastases or multiple high-risk factors were recommended to receive preoperative chemotherapy [13]. 5-fluorouracil/capecitabine combined with oxaliplatin/irinotecan were mainstay for chemotherapy regimen, with or without bevacizumab and cetuximab. Tumours were resected openly or laparoscopically.

### Follow-up and endpoints

Patients were followed up with every 3 months during the first 2 postoperative years and then every 6 months. The initial liver function test, serum CEA measurement, CT and MRI scans were performed one month after surgery. Clavien-Dindo classification system was applied to grade postoperative complications, defining I – II as minor complications and III—V as major complications [14]. From the date of hepatectomy, overall survival (OS) was calculated to the last follow-up or death and progression-free survival (PFS) was calculated to the last follow-up or tumour recurrence.

### Statistical analysis

The optimal cut-off values of AST, MLR, AMLRI, operation time and blood loss were determined at the point with the largest log-rank statistic by X-tile software (version 3.6.1, Yale University) according to OS. Patients were divided into high and low AMLRI groups according to the calculated cut-off value. Mann–Whitney U test were performed for the comparison of continuous parameters. For the categorical parameters, Pearson's chi-square test was applied. Time-dependent receiver operating characteristic (time-ROC) analysis was conducted and the area under the ROC curve (AUC) was estimated at the points of 1, 2, 3, 4, and 5 years after surgery, to evaluate the ability of the markers in predicting PFS and OS. Kaplan–Meier analysis was applied for survival analysis using the log-rank test. The prognostic values of each parameter for PFS and OS were assessed by univariable and multivariable Cox proportional hazards regression analysis. Parameters with *p* < 0.1 in the univariable analysis were included in the multivariable analysis by the forward stepwise (conditional LR) method. Nomograms for the probability of PFS and OS were established based on the results of the multivariable analysis, which were categorical. Additionally, nomograms whose variables were in the form of continuous variables rather than categorical ones were also established. Restricted cubic splines were used to permit nonlinear associations for continuous variables [15]. The performance of the nomograms was evaluated by calibration curves and concordance indexes (c-indexes) after bootstrapping with 1000 resamples. A larger c-index represented a more accurate prognostic ability of the nomogram. The results were compared to those of the CRS by c-indexes and time-ROC curves. Data were analyzed using SPSS (version 26.0, IBM) and R (version 4.0.5, R Core Team). All *p*-values were two-tailed, and *p* < 0.05 was considered statistically significant.

## Results

### Clinicopathologic characteristics of patients

Finally, 389 patients were included from November April 2012 to December 2018. Most patients were males, and their median age was 57 (interquartile range [IQR] 50–64) years. Extrahepatic metastases existed in 46 patients (12.1%) at the initial diagnosis. The primary tumours were in the colon in 204 (53.8%) patients, and 64 (16.9%) were situated in the right hemicolon. Synchronous liver metastasis was detected in 290 (76.5%) patients. A total of 203 (53.6%) patients had multiple metastases, and 133 (35.1%) of these metastases were distributed in both lobes. A total of 196 (51.7%) patients received preoperative chemotherapy. Hepatectomy was performed for all patients, among which 150 (39.6%) patients received major liver resection and 42 (11.1%) patients received radio frequency ablation (RFA). A total of 193 (50.9%) patients received postoperative chemotherapy. The median levels of AST, MLR and AMLRI, were 21 (IQR 16–27), 0.27 (IQR 0.20–0.36) and 5.67 (IQR 3.82–8.90), respectively.

### Optimal cut-off values and relationship between the AMLRI and clinicopathologic characteristics

The optimal cut-off values were identified by X-tile analysis by OS as follows: AST, 3.33; MLR, 0.15; AMLRI, 3.33; blood loss, 400; and operation time, 340. Patients were divided into low (≤ 3.33, *n* = 72) and high (> 3.33, *n* = 307) AMLRI groups. Patients in the high AMLRI group was observed with a higher BMI (*p* = 0.020), multiple metastases (*p* = 0.025), bilobar distribution (*p* = 0.011), high CRS (*p* = 0.029), major liver resection (*p* = 0.011), preoperative chemotherapy (*p* < 0.001), postoperative major complications (*p* = 0.043), and higher AST (*p* < 0.001) and MLR (*p* < 0.001). The detailed comparison of the two groups is shown in Table 1.

**Table 1:** Clinicopathologic characteristics of patients

Parameters	AMLRI ≤ 3.33 (n = 72)	AMLRI > 3.33 (*n* = 307)	All patients (*n* = 379)	*p* value
Age (years)	57(49.25–62)	57(51–65)	57(50–64)	0.378
Age ≥ 60 years	30 (41.7%)	126 (41.0%)	156 (41.2%)	0.923
Female	29 (40.3%)	115 (37.5%)	144 (38.0%)	0.657
BMI ≥ 24 kg/m^2^	28 (38.9%)	166 (54.1%)	194 (51.2%)	0.020^*^
ASA score 1–2	5 (6.9%)	36 (11.7%)	41 (10.8%)	0.240
Primary site colon	39 (54.2%)	165 (53.7%)	204 (53.8%)	0.949
Right hemicolon	13 (18.1%)	51 (16.6%)	64 (16.9%)	0.769
R0 resection	53 (73.6%)	206 (67.1%)	259 (68.3%)	0.285
Poorly differentiated	13 (18.1%)	86 (28.0%)	99 (26.1%)	0.083
T3-T4	69 (95.8%)	278 (90.6%)	347 (91.6%)	0.147
N1-N2	47 (65.3%)	217 (70.7%)	264 (69.7%)	0.369
Synchronous metastasis	58 (80.6%)	232 (75.6%)	290 (76.5%)	0.369
Extrahepatic metastases	13 (18.1%)	33 (10.7%)	46 (12.1%)	0.088
Diameter of metastases > 5 cm	6 (8.3%)	39 (12.7%)	45 (11.9%)	0.302
Multiple metastases	30 (41.7%)	173 (56.4%)	203 (53.6%)	0.025^*^
Bilobar distribution	16 (22.2%)	117 (38.1%)	133 (35.1%)	0.011^*^
Preoperative CEA > 50 ng/ml	10 (13.9%)	62 (20.2%)	72 (19.0%)	0.220
CRS 3–5	22 (30.6%)	137 (44.6%)	159 (42.0%)	0.029^*^
Major liver resection	19 (26.4%)	131 (42.7%)	150 (39.6%)	0.011^*^
Hepatectomy only	18 (25.0%)	112 (36.5%)	130 (34.3%)	0.065
Radio frequency ablation	5 (6.9%)	37 (12.1%)	42 (11.1%)	0.214
Preoperative chemotherapy	23 (31.9%)	173 (56.4%)	196 (51.7%)	< 0.001^*^
Postoperative chemotherapy	39 (54.2%)	154 (50.2%)	193 (50.9%)	0.541
Comorbidity	35 (48.6%)	136 (44.3%)	171 (45.1%)	0.508
Postoperative complication	36 (50.0%)	143 (46.6%)	179 (47.2%)	0.601
Postoperative major complication	7 (9.7%)	62 (20.2%)	68 (17.9%)	0.043^*^
Serious operation injury condition	24 (33.3%)	138 (45.0%)	162 (42.7%)	0.073
AMLRI	2.57 (1.87–3.01)	6.57 (4.92–10.09)	5.67 (3.82–8.90)	< 0.001^*^
AST (U/L)	16.00(13.25–18.00)	23(18–29)	21(16–27)	< 0.001^*^
MLR	0.16 (0.12–0.19)	0.29 (0.23–0.39)	0.27 (0.20–0.36)	< 0.001^*^
Blood loss (ml)	252.5 (180.0–332.5)	200 (100–400)	200 (100–400)	0.710
Operation time (min)	252.5 (180.00–332.5)	294 (184–378)	284 (180–370)	0.107

*BMI* body mass index, *ASA* American Society of Anesthesiologists *T* tumour staging, *N* lymph node staging, *CEA* carcinoembryonic antigen, *CRS* clinical risk score, *AMLRI* AST·MLR index, *AST* aspartate aminotransferase, *MLR* monocyte-to-lymphocyte ratio

^*^Statistically significant

### Prognostic values

**Fig. 1:**
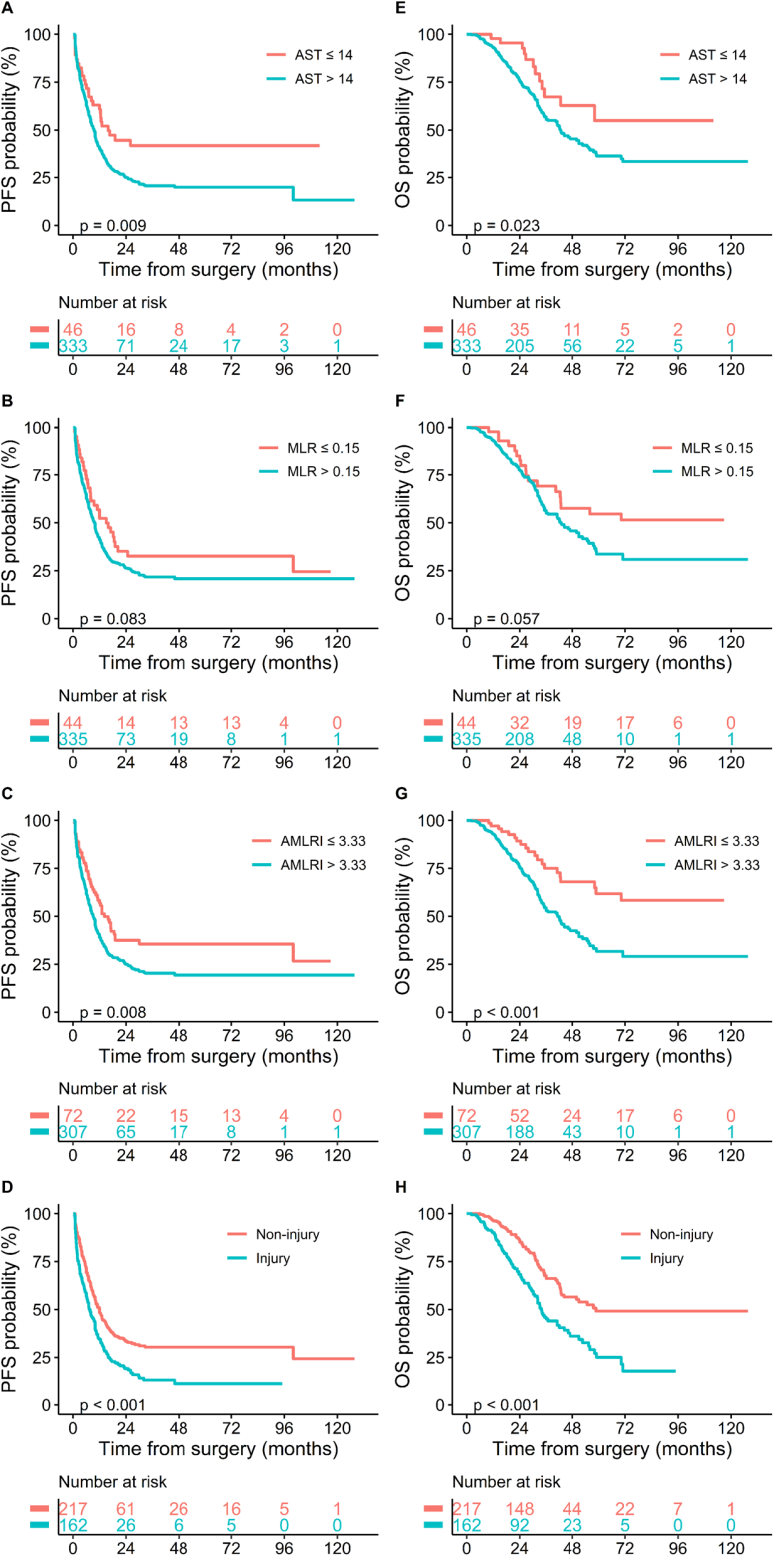
Kaplan–Meier curves for PFS (**A**–**D**) and OS (**E**–**H**) in CRLM patients stratified by AST, MLR, AMLRI and operation injury condition. CRLM, colorectal cancer liver metastases; AST, aspartate aminotransferase; MLR, monocyte-to-lymphocyte ratio; AMLRI, AST·MLR index

The median follow-up period was 43.4 months (IQR 40.6–46.2). Recurrence happened in 285 patients (75.20%) and 163 patients (43.01%) died. The median PFS was 10.0 months (IQR 8.6–11.4), and the median OS was 44.1 months (IQR 37.5–50.7). The 1-, 3- and 5-year PFS rates were 42.38%, 23.12%, and 22.48%, respectively. The 1-, 3- and 5-year OS rates were 93.60%, 57.00% and 38.41%, respectively. The Kaplan–Meier survival curves showed that patients with AST > 14 U/L (*p* = 0.009, median PFS: 9.5 vs. 15.9 months; *p* = 0.009, median OS: 47.6 vs. not-reached months), AMLRI > 3.33 (*p* = 0.008, median PFS: 9.1 vs. 14.2 months; *p* < 0.001, median OS: 41.2 vs. not-reached months) and serious operation injury condition (*p* < 0.001, median PFS: 7.5 vs. 12 months; *p* < 0.001, median OS: 33.8 vs. 58.8 months) presented significantly shorter PFS and OS, and patients with MLR > 0.15 presented the tendency of shorter PFS and OS (*p* = 0.083, median PFS: 9.9 vs. 15.0 months; *p* = 0.057, median OS: 42.7 vs. not-reached months) (Fig. 1).

### Time-ROC analysis

**Fig. 2:**
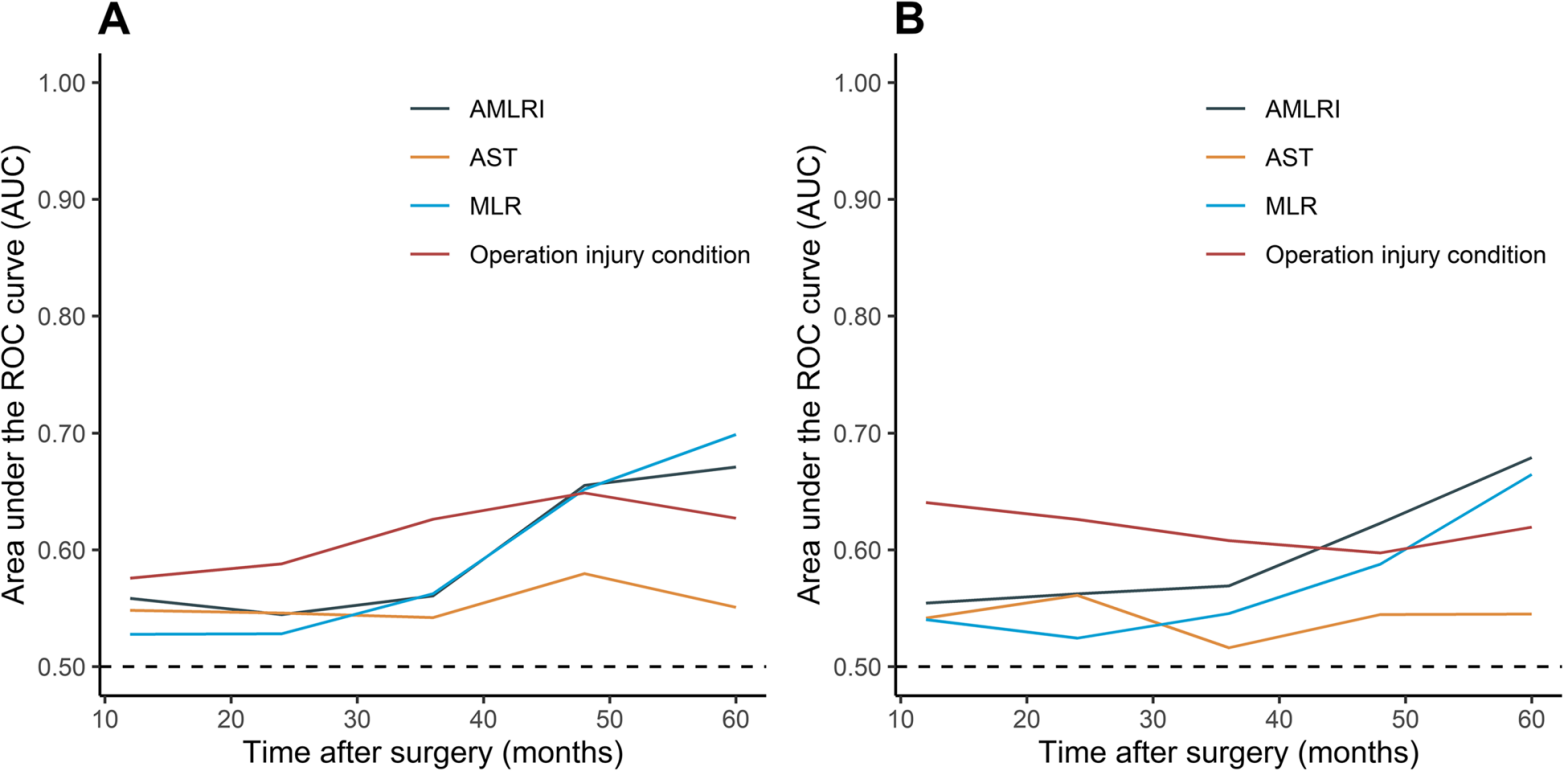
Time-dependent receiver operating characteristic (time-ROC) curves of AMLRI, AST, MLR and operation injury condition for PFS (**A**) and OS (**B**). AST, aspartate aminotransferase; MLR, monocyte-to-lymphocyte ratio; AMLRI, AST·MLR index

The AUCs of the AMLRI, AST, MLR and operation injury condition of the 1-, 2-, 3-, 4- and 5-year PFS and OS were calculated and presented by the time-ROC analysis (Fig. 2). For the OS, the AUCs of AMLRI and operation injury condition were continuously superior to that of AST and MLR. For the PFS, AUCs of operation injury condition were the highest before 5 years. The performance of AMLRI and MLR was similar, but the latter performed better for 5-year PFS. The detailed data were shown in S1 Table.

### Univariable and multivariable analyses for PFS

AMLRI > 3.33 was an independent risk factor for PFS according to the univariable Cox regression analysis (HR = 1.530, 95% confidence interval [CI]: 1.114–2.101, *p* = 0.009). Similar findings were observed for the AST > 14 U/L (HR = 1.697, 95% CI: 1.133–2.542, p = 0.010) and serious operation injury conditions (HR = 1.616, 95% CI: 1.279–2.042, *p* < 0.001). MLR > 0.15 presented limited prognostic value (HR = 1.397, 95% CI: 0.953–2.046, *p* < 0.086). Other risk predictors included R0 resection (*p* < 0.001), N1-N2 (*p* < 0.001), synchronous metastasis (*p* = 0.012), extrahepatic metastases (*p* = 0.004), multiple metastases (*p* < 0.001), bilobar distribution (*p* < 0.001), preoperative CEA > 50 ng/ml (*p* = 0.002), major liver resection (*p* < 0.001), RFA (*p* = 0.001), preoperative chemotherapy (*p* = 0.001), and postoperative complications (*p* = 0.005). AST and MLR were excluded from the multivariable analysis on account of their collinearity with the AMLRI, poor discriminative capacity of AST in time-ROC analysis and limited prognostic value of MLR in survival analysis. CRS was also excluded since it would be compared with this multivariable model. All other factors with *p* < 0.1 were included. AMLRI > 3.33 was still an independent risk factor (HR = 1.464, 95% CI: 1.060–2.022, *p* = 0.021) in this model. However, serious operation injury conditions were excluded from the model (*p* = 0.059). R0 resection (*p* < 0.001), N1-N2 (*p* = 0.004), extrahepatic metastases (*p* = 0.041), preoperative CEA > 50 ng/ml (*p* = 0.013) and major liver resection (*p* < 0.001) were also included (Table 2).

**Table 2:** Univariable and multivariable analysis of parameters for progression-free survival

Parameters	Univariable analysis			Multivariable analysis		
	HR	95% CI	*P* value	HR	95% CI	*P* value
Age ≥ 60 years	0.993	0.783—1.260	0.957			
Female	0.957	0.752—1.217	0.719			
BMI ≥ 24 kg/m^2^	0.891	0.706—1.124	0.328			
ASA score 1–2	1.051	0.724—1.526	0.794			
Primary site colon	1.052	0.833—1.330	0.669			
Right hemicolon	1.066	0.781—1.453	0.688			
R0 resection^a^	0.515	0.404—0.658	< 0.001^*^	0.583	0.453—0.748	< 0.001^*^
Poorly differentiated^a^	1.286	0.990—1.671	**0.060**			0.684
T3-T4^a^	1.490	0.935—2.374	**0.093**			0.262
N1-N2^a^	1.749	1.332—2.295	< 0.001^*^	1.511	1.145—1.994	0.004^*^
Synchronous metastasis^a^	1.451	1.087—1.937	0.012^*^			0.143
Extrahepatic metastases^a^	1.612	1.160—2.239	0.004^*^	1.416	1.015—1.976	0.041^*^
Diameter of metastases > 5 cm	1.038	0.722—1.492	0.841			
Multiple metastases^a^	1.997	1.571—2.539	< 0.001^*^			0.063
Bilobar distribution^a^	2.035	1.603—2.583	< 0.001^*^			0.115
Preoperative CEA > 50 ng/ml^a^	1.569	1.183—2.082	0.002^*^	1.400	1.053—1.861	0.021^*^
Major liver resection^a^	1.978	1.563—2.503	< 0.001^*^	1.615	1.266—2.060	< 0.001^*^
Hepatectomy only	0.869	0.678—1.113	0.267			
Radio frequency ablation^a^	1.818	1.289—2.565	0.001^*^			0.153
Preoperative chemotherapy^a^	1.481	1.170—1.874	0.001^*^			0.204
Postoperative chemotherapy	0.870	0.689—1.098	0.241			
Comorbidity	0.960	0.760—1.213	0.731			
Postoperative complication^a^	1.393	1.103—1.757	0.005^*^			0.161
Postoperative major complication	1.120	0.827—1.517	0.463			
Serious operation injury condition^a^	1.616	1.279—2.042	< 0.001^*^			0.062
AMLRI > 3.33^a^	1.530	1.114—2.101	0.009^*^	1.462	1.059—2.019	0.021 ^*^
AST > 14 U/L	1.697	1.133—2.542	0.010^*^			
MLR > 0.15	1.397	0.953—2.046	0.086			
Blood loss (ml)	1.521	1.157—1.999	0.003^*^			
Operation time (min)	1.572	1.240—1.993	< 0.001^*^			
CRS 3–5	1.608	1.272—2.032	< 0.001^*^			

*BMI* body mass index, *ASA* American Society of Anesthesiologists *T* tumour staging, *N* lymph node staging, *CEA* carcinoembryonic antigen, *CRS* clinical risk score, *AMLRI* AST·MLR index, *AST* aspartate aminotransferase, *MLR* monocyte-to-lymphocyte ratio

^a^Included in multivariable analysis

*Statistically significant

### Univariable and multivariable analyses for OS

AMLRI > 3.33 (HR = 2.295, 95% CI: 1.430–3.684, *p* = 0.001), AST > 0.15 U/L (HR = 1.948, 95% CI: 1.082–3.508, *p* = 0.026) and serious operation injury condition (HR = 2.072, 95% CI: 1.520–2.824, *p* < 0.001) were also independent risk factors in the OS analysis. Other risk predictors included R0 resection (*p* < 0.001), N1-N2 (*p* = 0.002), extrahepatic metastases (*p* = 0.007), multiple metastases (*p* < 0.001), bilobar distribution (*p* < 0.001), preoperative CEA > 50 ng/ml (*p* = 0.004), major liver resection (*p* < 0.001), preoperative chemotherapy (*p* = 0.003), postoperative chemotherapy (*p* = 0.001) and postoperative complications (*p* < 0.001). Similarly, AST and MLR were excluded for the same reason in PFS analysis. CRS was also excluded since it would be compared with this multivariable model. All other factors with *p* < 0.1 were included. AMLRI > 3.33 (HR = 2.181, 95% CI: 1.345–3.538, *p* = 0.002) and serious operation injury condition (HR = 1.547, 95% CI: 1.108–2.162, *p* = 0.010) were still independent risk factors in the multivariable analysis. R0 resection (*p* = 0.009), N1-N2 (*p* = 0.042), extrahepatic metastases (*p* = 0.017), preoperative CEA > 50 ng/ml (*p* = 0.017), major liver resection (*p* = 0.090), postoperative chemotherapy (*p* = 0.001) and postoperative complications (*p* = 0.007) were also included in the model (Table 3).

**Table 3:** Univariable and multivariable analysis of parameters for overall survival

Parameters	Univariable analysis			Multivariable analysis		
	HR	95% CI	*P* value	HR	95% CI	*P* value
Age ≥ 60 years	1.028	0.749—1.412	0.863			
Female	0.872	0.637—1.194	0.394			
BMI ≥ 24 kg/m^2^	0.981	0.721—1.333	0.901			
ASA score 1–2	1.212	0.766—1.917	0.412			
Primary site colon	1.186	0.870—1.618	0.281			
Right hemicolon	1.162	0.778—1.736	0.464			
R0 resection^a^	0.506	0.368—0.694	< 0.001^*^	0.635	0.455—0.887	0.008^*^
Poorly differentiated	1.087	0.758—1.559	0.649			
T3-T4	1.182	0.656—2.128	0.578			
N1-N2^a^	1.798	1.231—2.626	0.002^*^	1.512	1.027—2.225	0.036^*^
Synchronous metastasis	1.388	0.929—2.075	0.109			
Extrahepatic metastases^a^	1.804	1.176—2.768	0.007^*^	1.705	1.100—2.641	0.017^*^
Diameter of metastases > 5 cm	0.925	0.560—1.529	0.762			
Multiple metastases^a^	1.814	1.316—2.501	< 0.001^*^			0.221
Bilobar distribution^a^	1.899	1.386—2.602	< 0.001^*^			0.663
Preoperative CEA > 50 ng/ml^a^	1.703	1.190—2.437	0.004^*^	1.500	1.039—2.166	0.030^*^
Major liver resection^a^	2.159	1.582—2.948	< 0.001^*^	1.353	0.962—1.903	0.082^**^
Hepatectomy only	0.969	0.701—1.339	0.848			
Radio frequency ablation^a^	1.523	0.986—2.354	0.058			0.764
Preoperative chemotherapy^a^	1.630	1.177—2.258	0.003^*^			0.856
Postoperative chemotherapy^a^	0.605	0.444—0.825	0.001^*^	0.591	0.432—0.809	0.001^*^
Comorbidity	0.907	0.664—1.240	0.542			
Postoperative complication^a^	1.832	1.343—2.498	< 0.001^*^	1.552	1.125—2.142	0.007^*^
Postoperative major complication	1.218	0.810—1.833	0.344			
Serious operation injury condition^a^	2.072	1.520—2.824	< 0.001^*^	1.539	1.101—2.151	0.012^*^
AMLRI > 3.33^a^	2.295	1.430—3.684	0.001^*^	2.162	1.334—3.503	0.002^*^
AST > 14 U/L	1.948	1.082—3.508	0.026^*^			
MLR > 0.15	1.618	0.982—2.667	0.059			
Blood loss (ml)	1.969	1.399—2.771	< 0.001^*^			
Operation time (min)	1.596	1.170—2.177	< 0.001^*^			
CRS 3–5	1.788	1.312—2.436	< 0.001^*^			

*BMI* body mass index, *ASA* American Society of Anesthesiologists *T* tumour staging, *N* lymph node staging, *CEA* carcinoembryonic antigen, *CRS* clinical risk score, *AMLRI* AST·MLR index, *AST* aspartate aminotransferase, *MLR* monocyte-to-lymphocyte ratio

^a^Included in multivariable analysis

^*^Statistically significant

**0.05 < p < 0.10, included automatically by the forward stepwise (conditional LR) method of Cox regression

### Nomogram and performance

**Fig. 3:**
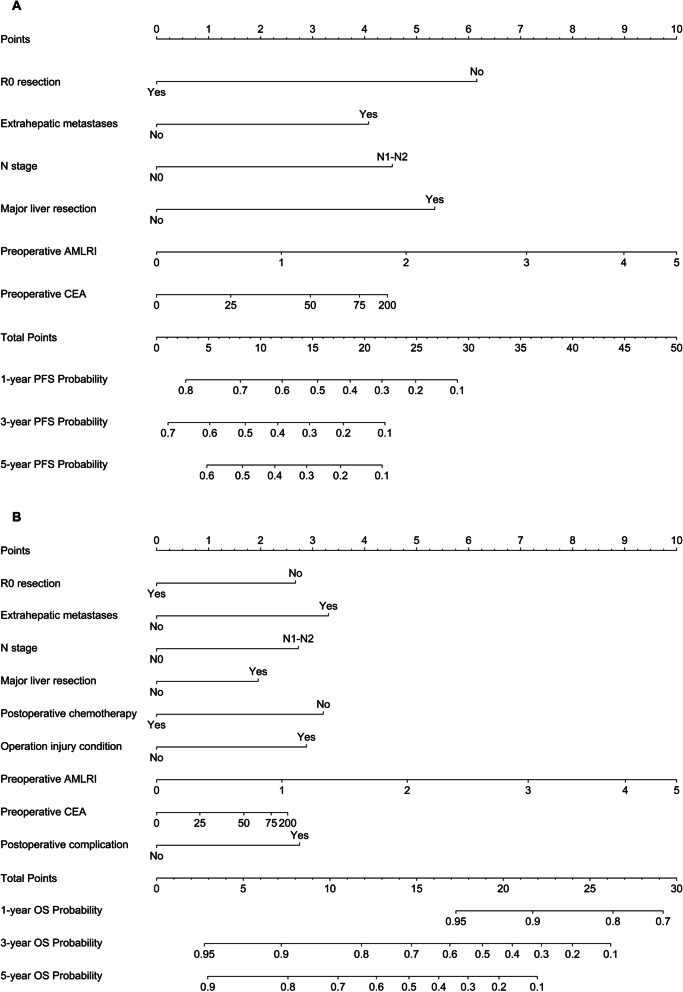
Nomograms for survival. **A** Nomogram for OS; (**B**) nomogram for PFS. The sum of the scores for each variable is plotted on the total points axis; the estimated probabilities of PFS or OS at 1-, 3- and 5- years were obtained by drawing a line perpendicularly from the plotted total points axis straight to the survival axis. AST, aspartate aminotransferase; MLR, monocyte-to-lymphocyte ratio; AMLRI, AST·MLR index; CEA, carcinoembryonic antigen

**Fig. 4:**
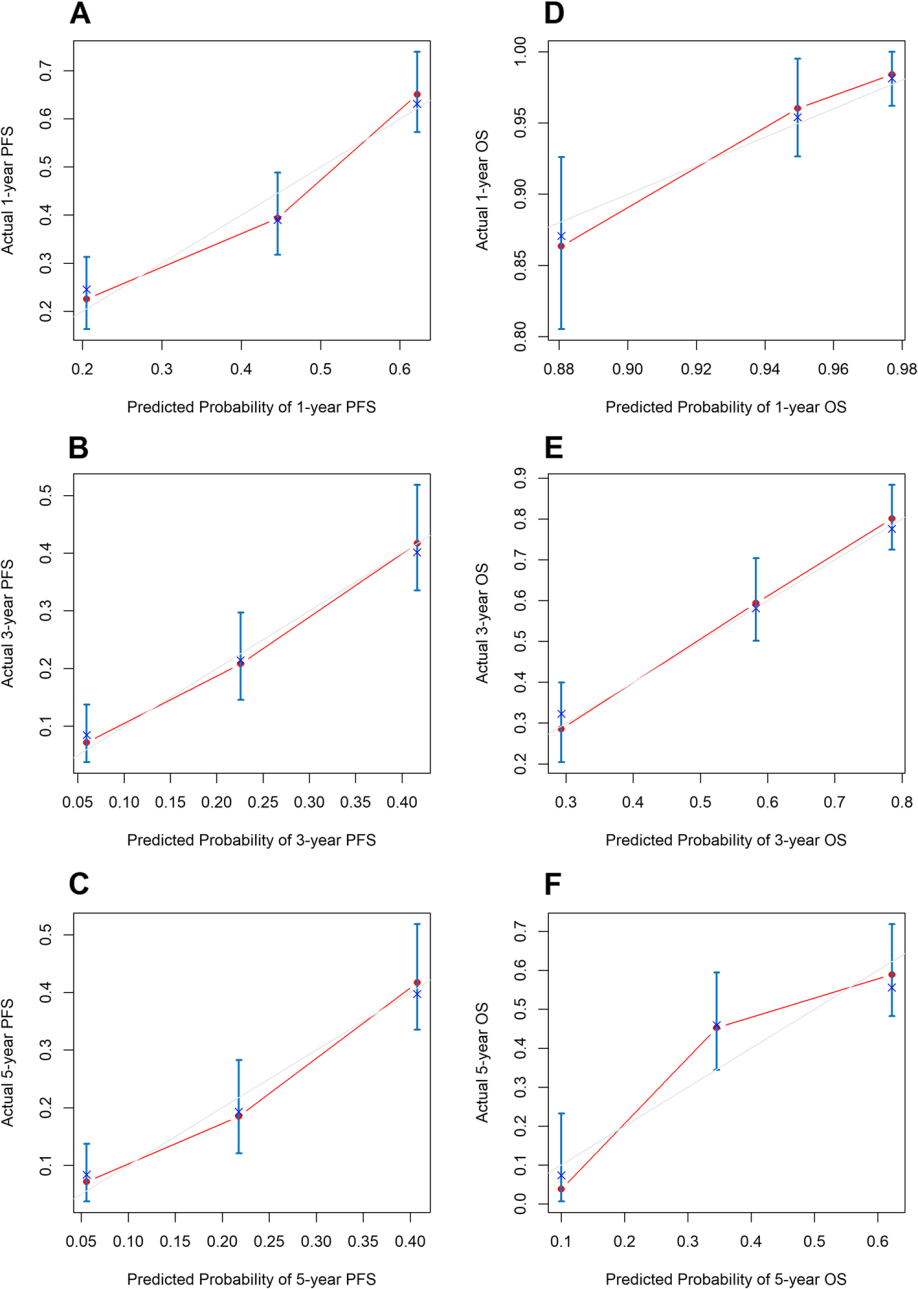
Calibration curves for predicting 1-year (**A**), 3-year (**B**) and 5-year (**C**) PFS and 1-year (**D**), 3-year (**E**) and 5-year (**F**) OS. Predicted survival produced by the nomogram is plotted on the x-axis, and actual survival is plotted on the y-axis. Dashed lines represent an identical calibration model in which the predicted PFS or OS approximate the actual PFS or OS

**Fig. 5:**
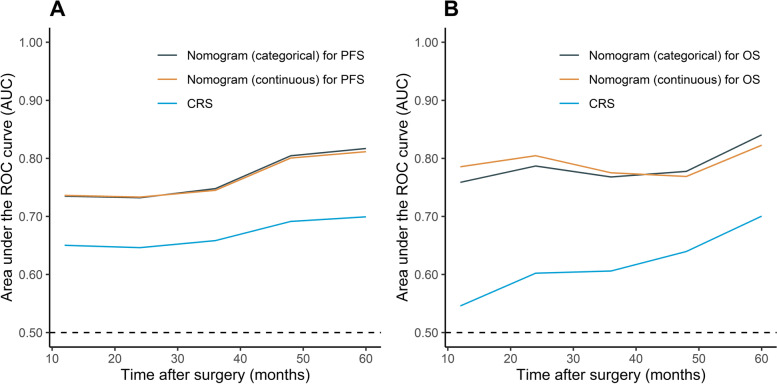
Time-dependent receiver operating characteristic (time-ROC) curves of established new models and clinical risk score for PFS (**A**) and OS (**B**). CRS, clinical risk score

The prognostic nomograms for PFS and OS (S1 Figure and S2-S3 Table) using categorical variables were developed according to the results of multivariable Cox models. For the nomograms using continuous variables, restricted cubic splines were applied to permit nonlinear associations (Fig. 3). A score was assigned to each predictor in the nomogram (top scale). The sum of these scores represented the probability of 1-, 3- and 5-year survival (bottom scale). In addition, the corresponding calibration curves of these nomograms were displayed, presenting a good result (Fig. 4, S2 Figure). The c-indexes for the categorical nomograms of PFS and OS were 0.653 and 0.731, respectively. The c-indexes for the categorical nomograms of PFS and OS were 0.653 and 0.726, respectively. The c-index of Fong’s CRS of PFS and OS were 0.600 and 0.586, respectively. The comparison with CRS was also performed using time-ROC, in which the new models performed better in both PFS and OS prediction (Fig. 5). The new models achieved fine to good discriminative capacity. The detailed data was contained in S4 Table.

## Discussion

Here, we first evaluated whether preoperative AST, MLR and their derivative AMLRI were of prognostic significance in patients with CRLM undergoing hepatectomy, and furtherly compared their ability of prediction by time- ROC curves. We also explored the prognostic significance of the operation injury condition, which combines operation time and blood loss. Then we developed two novel nomograms which have achieved greater performance than CRS, especially for OS prediction.

There is increasing evidence showing the inflammation is of importance in the development and progression of cancers [5, 16]. We mainly focused on the MLR. Previous studies about the MLR focused on patients with unresectable tumours receiving radiofrequency ablation or patients with liver-only resectable tumours receiving neoadjuvant therapy [6, 7]. The population of these studies were limited, and we included all patients undergoing hepatectomy regardless of whether there were extrahepatic metastases or neoadjuvant therapy. The MLR successfully showed a limited prognostic value (*p* < 0.1). The possible mechanisms could be associated with the functions of monocytes and monocyte-derived tumour-associated macrophages (TAMs). These cells make a crucial contribution to tumour growth, invasion, angiogenesis, immunosuppression and resistance against chemotherapy and radiotherapy [17]. For example, epithelial-mesenchymal transition (EMT) is often activated in metastatic tumours during tumour invasion and metastasis. TAM infiltration is associated with EMT marker expression (Snail, E-cadherin and Vimentin) [18]. The process of EMT to enhance CRC migration, invasion, and metastasis can conversely lead to the production of CCL2, which promotes macrophage recruitment [19]. These interaction between inflammatory cells and other cells and tissues might eventually lead to tumour progression.

AST has been routinely used as a liver function test index. It is a good all-cause and liver-related mortality predictor [20]. Some studies have evaluated its prognostic value in solid tumours, but mostly in the form of a ratio with other parameters, such as platelet counts, neutrophil counts, and alanine aminotransferase level [3, 4]. A study in patients with non-small cell lung cancer showed that the AST level could predict PFS and OS [21]. It was also demonstrated in our study that a higher AST level indicates a poorer prognosis, which was first reported in CRLM. The association between AST level and cancer prognosis is unclear. The most likely explanation focuses on the metabolic mechanism of cancer cells. Some studies have shown that cancer cells can obtain energy through glutamine metabolism catalyzed by AST [22]. Another study reported that AST inhibitors could inhibit the proliferation of transformed breast adenocarcinoma cells in vitro by inhibiting tricarboxylic acid cycle [23]. In conclusion, current studies do not provide a convincing mechanism to explain the role of AST in tumour development.

We noticed that the AST cut-off value in this study was below the standard range of our hospital. Possible explanations might be related to the clinical application of AST. It is mainly used to diagnose liver diseases such as cirrhosis. In other words, the standard reference range was determined to evaluate other kinds of diseases rather than cancer or, to be more specific, CRLM. CRLM is essentially a colorectal disease that metastasizes to the liver; therefore, it is natural that these patients present with different levels of AST than the levels seen in other diseases. The AST cut-off value was also below the standard reference range in Chen et al.’s study focusing on the prognostic value of AST in lung cancer [21]. CRLM patients with AST levels exceeding the cut-off value determined in our study might be at risk for poor postoperative prognosis, but they are still within the normal range for evaluating liver function.

Multiplying AST by MLR, the AMLRI was defined. AST performed well in the Kaplan–Meier analysis; however, the results of time-ROC was poor. The MLR presented limited prognostic value in the Kaplan–Meier analysis, but in the time-ROC it was much better than AST. Therefore, the two markers were combined and inherited both merits with significant results in Kaplan–Meier, time-ROC, and univariable Cox regression analysis, and furtherly successfully proved to be a good prognostic predictor and included into the final nomograms.

The operation injury condition was an exploratory parameter that was proposed to assess the injury level resulting from the operation. Both operation time and blood loss are direct reflection of the injury which patients sustained during operation. They presented excellent prognostic value in our univariable Cox analysis. After combined, the operation injury condition performed incredibly in Kaplan–Meier and time-ROC analysis with high prognostic value and discriminative capacity. Furthermore, it was included in the multivariable Cox model for OS. It was excluded from the PFS model, as we noticed that the operation injury condition had a p-value of 0.062. Theoretically, longer operation times and greater blood loss are considered markers of increased surgery-related injury, and surgery might influence the prognosis of cancer after curative resection by activating the stress response because of surgery or anaesthesia [24].

As discussed previously, inflammation plays a crucial role in the progression of cancer. In addition, although the evidence is currently inconclusive, it is understandable exist that tumour cells released in the form of circulating tumour cells during surgery might lead to metastatic colonization [25]. The surgical stress response due to the activation of the sympathetic nervous system, might also induce cancer recurrence. The effects of perioperatively increased catecholamines and prostaglandins on cancer progression have been widely studied in animal models [26]. A study in athymic nude mice with tumour cell injection showed that weight and size of tumour nodules of the mice receiving laparotomy and mastectomy were significantly greater than anaesthesia-only ones, and β-adrenoceptor blockade could prevent such effects [27]. Some randomized controlled trials are in progress, and in a study for colorectal cancer, patients who received perioperative COX2 and β-adrenergic blockade showed a significant improvement in tumour molecular markers, and the 3-year recurrence rates were also lower (in protocol-compliant patients: 0/11 compared with 5/17) [28]. Psychological stress, immunosuppression resulting from other hormones, such as glucocorticoids, and anaesthetic choice are also associated with this process [29].

Fong’s clinical risk score has been widely used since its publication to predict recurrence and survival for patients with CRLM [10]. Humans have achieved a more profound understanding of the progression mechanism of cancer, such as the role of inflammation and metabolism; thus, more risk factors should be integrated into the prediction model to help medical teams choose therapeutic strategies and judge prognosis. After combining the AMLRI and operation injury condition with some patient clinicopathologic characteristics, we successfully developed novel nomograms for predicting the PFS and OS of patients with CRLM undergoing hepatectomy by using both categorical and continuous variables, which could be chosen according to situation. The nomograms had higher accuracy than Fong’s clinical risk score system according to the c-indexes. Our model is comprehensive, as it consists of preoperative serum markers, surgery conditions and tumour biological characteristics that are all easily accessible for every patient; thus, the model is applicable to a broader population of patients. This is the first study to integrate the inflammation marker AMLRI and operation injury condition into the prediction model, and all nomograms achieved good c-index, time-ROC, and calibration curve results. It is worth noting that Fong et al. developed a nomogram based on Fong’s clinical risk score [30]. But we are unable to compare Fong’s nomogram with ours, since there is no detailed record in our database currently for some variables they used. Further studies are needed for the comparison to improve the performance of our nomogram.

Limitations also existed in this study. First, the data were retrospectively collected, which may introduce bias. Second, blood samples were collected only once before surgery, and the values may have fluctuated during the perioperative period. The postoperative and the perioperative dynamic changes could be evaluated in the future. Third, as a single-centre study, the lack of external validation may reduce the generalizability to other populations. Fourth, genetic conditions such as KRAS and BRAF were not included in the analysis. Since genetic testing is not covered by health insurance, many patients do not receive it, which leads to a lack of related information. Genetic conditions are powerful prognostic factors in predicting survival of CRLM patients after resection. By combining RAS status, Brudvik et al. modified the traditional CRS. The new risk score contained only 3 factors and performed better than the traditional CRS, which represented the importance of RAS status [31]. If the genetic conditions could be included, the new nomogram might perform better. However, as many patients lack the data of genetic conditions in China, the application of a model including them might be limited. A multi-centre study could be conducted to reduce bias and deficiency of data. Fifth, operation injury condition might be of some prognostic value, but it needs to be noted that numerous confounding factors are involved, as discussed below. Defining injury condition by only two factors, operation time and blood loss, was relatively non-specific. More factors should be considered in future study such as blood transfusion and types of anaesthetics.

## Conclusion

The AMLRI, which combines AST and MLR, is an easily accessible preoperative biomarker that accurately predicts PFS and OS in patients with CRLM undergoing hepatectomy. The operation injury condition was also predictive for OS. The novel nomograms based on these two markers performed better than Fong’s CRS for predicting recurrence and survival.

## Supplementary Information

**Additional file 1: S1 Figure.** Nomograms (categorical) for survival. (A) Nomogram for PFS; (B) nomogram for OS. The sum of the scores for each variable is plotted on the total points axis; the estimated probabilities of PFS or OS at 1-, 3- and 5- years were obtained by drawing a line perpendicularly from the plotted total points axis straight to the survival axis. PFS, progression-free survival; OS, overall survival; AST, aspartate aminotransferase; MLR, monocyte-to-lymphocyte ratio; AMLRI, AST·MLR index; CEA, carcinoembryonic antigen. **S2 Figure.** Calibration curves for predicting 1-year (A), 3-year (B) and 5-year (C) PFS and 1-year (D), 3-year (E) and 5-year (F) OS. Predicted survival produced by the nomogram (categorical) is plotted on the x-axis, and actual survival is plotted on the y-axis. Dashed lines represent an identical calibration model in which the predicted PFS or OS approximate the actual PFS or OS. PFS, progression-free survival; OS, overall survival. **S1 Table.** AUCs of the markers. **S2 Table.** Prognostic score of the nomograms for progression-free survival. **S3 Table.** Prognostic score of the nomograms for overall survival. **S4 Table.** C-indexes and AUCs of the nomograms and Fong’s clinical risk score.

## Data Availability

The datasets generated during the current study are not publicly available due to the hospital regulations, but are available from the corresponding author on reasonable request.

## Abbreviations

AMLRI: AST·MLR index
ASA: American Society of Anesthesiologists
AST: Aspartate aminotransferase
AUC: Area under the ROC curve
BMI: Body mass index
CEA: Carcinoembryonic antigen
CI: Confidence interval
CRC: Colorectal cancer
CRLM: Colorectal cancer liver metastases
CRS: Clinical risk score
HR: Hazard ratio
IQR: Interquartile range
MLR: Monocyte-to-lymphocyte ratio
OS: Overall survival
PFS: Progression-free survival
RFA: Radio frequency ablation
ROC: Receiver operating characteristic curve
time-ROC: Time-dependent receiver operating characteristic
