# Supplementary material for: AST·MLR index and operation injury condition are novel prognostic predictor for the prediction of survival in patients with colorectal cancer liver metastases undergoing surgical resection
Source: BMC Cancer. 2022 Aug 26;22:921. doi: 10.1186/s12885-022-10009-4 (PMC9414420; doi:10.1186/s12885-022-10009-4)
Supplement: Supplementary file 1 — Additional file 1: S1 Figure. Nomograms (categorical) for survival. (A) Nomogram for PFS; (B) nomogram for OS. The sum of the scores for each variable is plotted on the total points axis; the estimated probabilities of PFS or OS at 1-, 3- and 5- years were obtained by drawing a line perpendicularly from the plotted total points axis straight to the survival axis. PFS, progression-free survival; OS, overall survival; AST, aspartate aminotransferase; MLR, monocyte-to-lymphocyte ratio; AMLRI, AST·MLR index; CEA, carcinoembryonic antigen. S2 Figure. Calibration curves for predicting 1-year (A), 3-year (B) and 5-year (C) PFS and 1-year (D), 3-year (E) and 5-year (F) OS. Predicted survival produced by the nomogram (categorical) is plotted on the x-axis, and actual survival is plotted on the y-axis. Dashed lines represent an identical calibration model in which the predicted PFS or OS approximate the actual PFS or OS. PFS, progression-free survival; OS, overall survival. S1 Table. AUCs of the markers. S2 Table. Prognostic score of the nomograms for progression-free survival. S3 Table. Prognostic score of the nomograms for overall survival. S4 Table. C-indexes and AUCs of the nomograms and Fong’s clinical risk score. [file 12885_2022_10009_MOESM1_ESM.docx]

**S1 Figure**


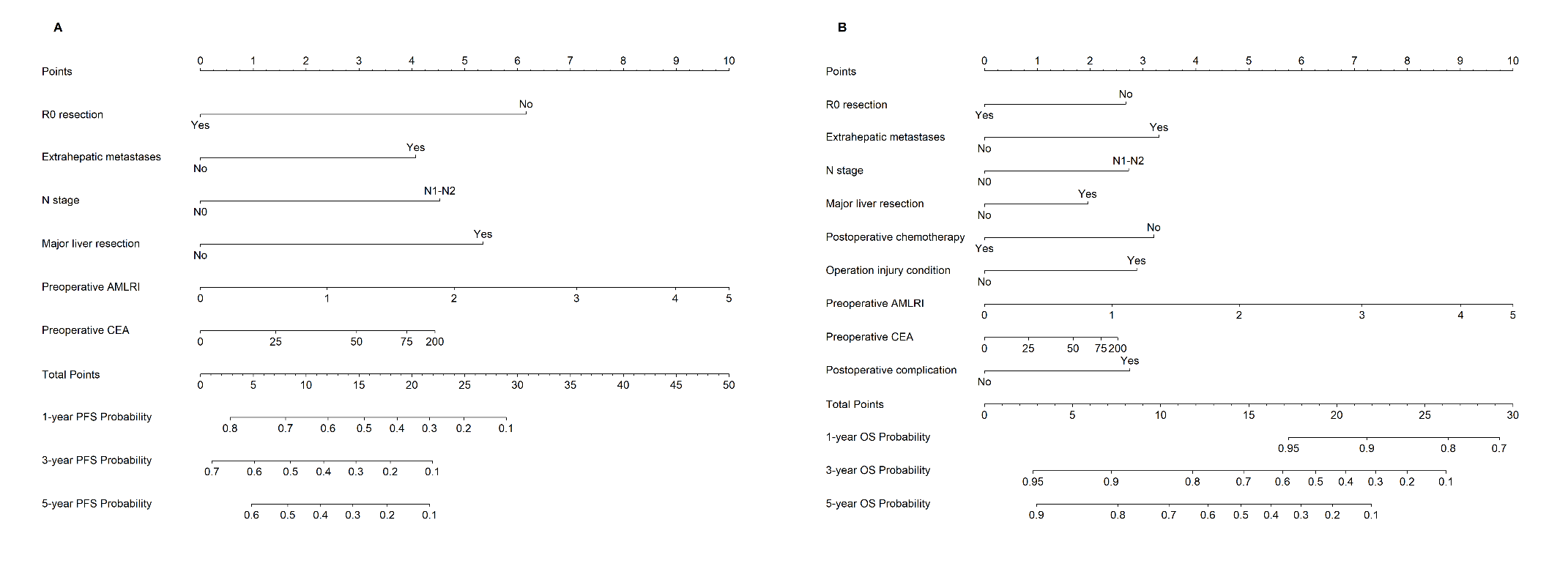


Nomograms (categorical) for survival. (A) Nomogram for PFS; (B) nomogram for OS. The sum of the scores for each variable is plotted on the total points axis; the estimated probabilities of PFS or OS at 1-, 3- and 5- years were obtained by drawing a line perpendicularly from the plotted total points axis straight to the survival axis. PFS, progression-free survival; OS, overall survival; AST, aspartate aminotransferase; MLR, monocyte-to-lymphocyte ratio; AMLRI, AST·MLR index; CEA, carcinoembryonic antigen.

**S2 Figure**


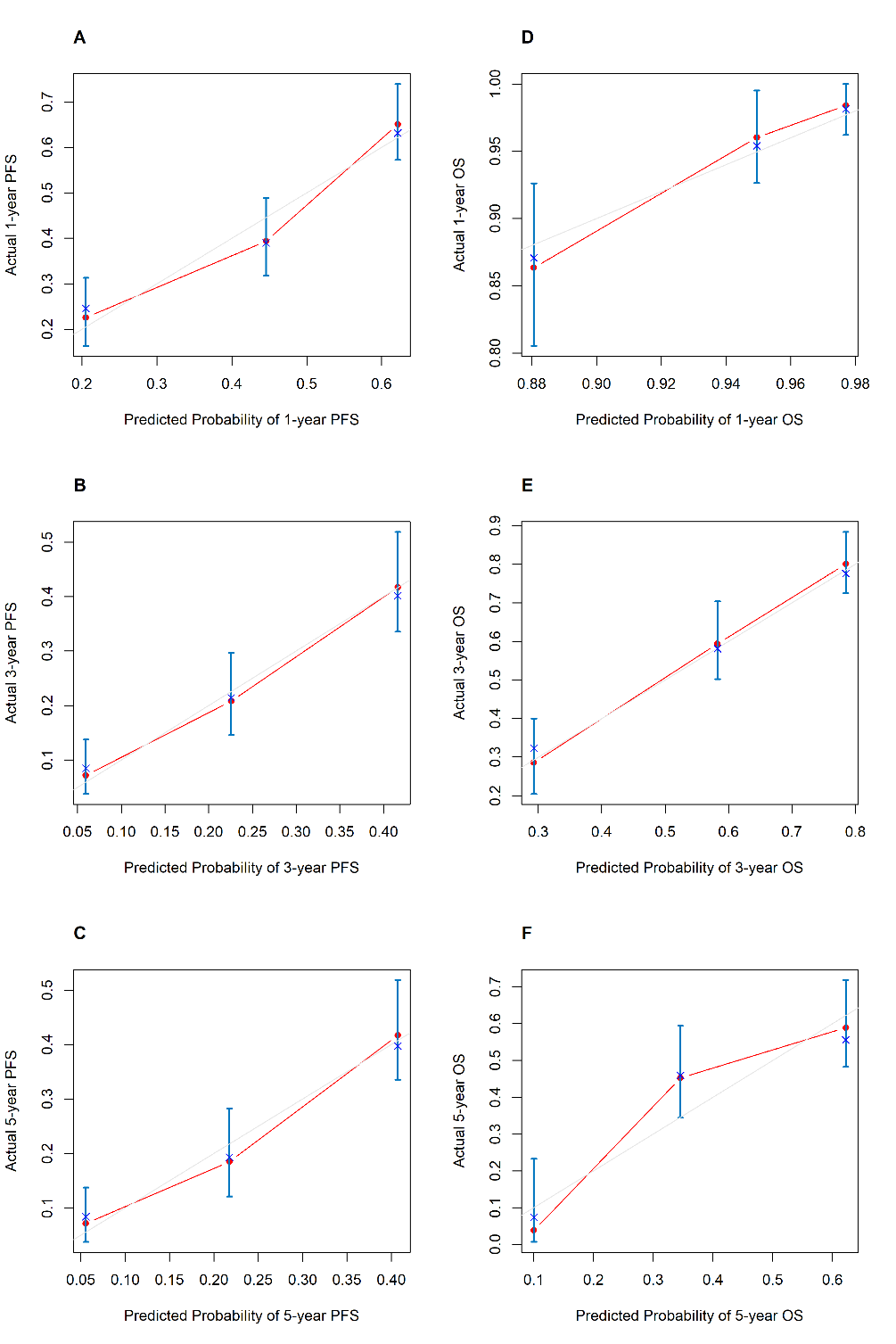


Calibration curves for predicting 1-year (A), 3-year (B) and 5-year (C) PFS and 1-year (D), 3-year (E) and 5-year (F) OS. Predicted survival produced by the nomogram (categorical) is plotted on the x-axis, and actual survival is plotted on the y-axis. Dashed lines represent an identical calibration model in which the predicted PFS or OS approximate the actual PFS or OS. PFS, progression-free survival; OS, overall survival.

**S1 Table AUCs of the markers**

|  | Parameters | AUC | | | |  |
| --- | --- | --- | --- | --- | --- | --- |
|  |  | 1 year | 2 year | 3 year | 4 year | 5 year |
| PFS | AMLRI | 0.558 | 0.545 | 0.561 | 0.655 | 0.671 |
|  | AST | 0.548 | 0.546 | 0.542 | 0.580 | 0.551 |
|  | MLR | 0.528 | 0.528 | 0.563 | 0.652 | 0.699 |
|  | Serious operation injury condition | 0.576 | 0.588 | 0.626 | 0.649 | 0.627 |
| OS | AMLRI | 0.554 | 0.562 | 0.569 | 0.623 | 0.679 |
|  | AST | 0.542 | 0.561 | 0.516 | 0.545 | 0.545 |
|  | MLR | 0.540 | 0.524 | 0.545 | 0.588 | 0.665 |
|  | Serious operation injury condition | 0.641 | 0.626 | 0.608 | 0.597 | 0.619 |

PFS, progression-free survival; OS, overall survival; AST, aspartate aminotransferase; MLR, monocyte-to-lymphocyte ratio; AMLRI, AST·MLR index.

**S2 Table Prognostic score of the nomograms for progression-free survival**

| Parameters | Score |
| --- | --- |
| R0 resection | 10.0 |
| Extrahepatic metastases | 6.5 |
| N1-N2 | 7.6 |
| Major liver resection | 8.8 |
| Preoperative AMLRI>3.33 | 7.1 |
| Preoperative CEA > 50 ng/ml | 6.7 |

AMLRI, AST·MLR index; CEA, carcinoembryonic antigen.

**S3 Table Prognostic score of the nomograms for overall survival**

| Parameters | Score |
| --- | --- |
| R0 resection | 5.7 |
| Extrahepatic metastases | 6.9 |
| N1-N2 | 5.2 |
| Major liver resection | 3.8 |
| Postoperative chemotherapy | 6.7 |
| Operation injury condition | 5.6 |
| Preoperative AMLRI>3.33 | 10.0 |
| Preoperative CEA > 50 ng/ml | 5.8 |
| Postoperative complication | 5.7 |

AMLRI, AST·MLR index; CEA, carcinoembryonic antigen.

**S4 Table C-indexes and AUCs of the nomograms and Fong’s clinical risk score**

|  | Parameters | C-index | AUC | | | |  |
| --- | --- | --- | --- | --- | --- | --- | --- |
|  |  |  | 1 year | 2 year | 3 year | 4 year | 5 year |
| PFS | Nomogram (categorical) | 0.653 | 0.735 | 0.732 | 0.748 | 0.804 | 0.817 |
|  | Nomogram (continuous) | 0.653 | 0.736 | 0.733 | 0.745 | 0.801 | 0.812 |
|  | CRS | 0.600 | 0.650 | 0.646 | 0.658 | 0.691 | 0.699 |
| OS | Nomogram (categorical) | 0.731 | 0.759 | 0.787 | 0.768 | 0.778 | 0.840 |
|  | Nomogram (continuous) | 0.726 | 0.786 | 0.805 | 0.775 | 0.769 | 0.823 |
|  | CRS | 0.586 | 0.546 | 0.602 | 0.606 | 0.640 | 0.701 |

PFS, progression-free survival; OS, overall survival; CRS, clinical risk score.
